# Effects of a Web-Based Tailored Multiple-Lifestyle Intervention for Adults: A Two-Year Randomized Controlled Trial Comparing Sequential and Simultaneous Delivery Modes

**DOI:** 10.2196/jmir.3094

**Published:** 2014-01-27

**Authors:** Daniela N Schulz, Stef PJ Kremers, Corneel Vandelanotte, Mathieu JG van Adrichem, Francine Schneider, Math JJM Candel, Hein de Vries

**Affiliations:** ^1^CAPHRI School for Public Health and Primary CareDepartment of Health PromotionMaastricht UniversityMaastrichtNetherlands; ^2^Nutrition and Toxicology Research Institute Maastricht (NUTRIM)Department of Health PromotionMaastricht UniversityMaastrichtNetherlands; ^3^Institute for Health and Social Science ResearchCentre for Physical Activity StudiesCentral Queensland UniversityRockhamptonAustralia; ^4^CAPHRI School for Public Health and Primary CareDepartment of Methodology and StatisticsMaastricht UniversityMaastrichtNetherlands

**Keywords:** multiple behavior change, Web-based intervention, computer tailoring, effectiveness, physical activity, fruit consumption, vegetable consumption, alcohol intake, smoking

## Abstract

**Background:**

Web-based computer-tailored interventions for multiple health behaviors can have a significant public health impact. Yet, few randomized controlled trials have tested this assumption.

**Objective:**

The objective of this paper was to test the effects of a sequential and simultaneous Web-based tailored intervention on multiple lifestyle behaviors.

**Methods:**

A randomized controlled trial was conducted with 3 tailoring conditions (ie, sequential, simultaneous, and control conditions) in the Netherlands in 2009-2012. Follow-up measurements took place after 12 and 24 months. The intervention content was based on the I-Change model. In a health risk appraisal, all respondents (N=5055) received feedback on their lifestyle behaviors that indicated whether they complied with the Dutch guidelines for physical activity, vegetable consumption, fruit consumption, alcohol intake, and smoking. Participants in the sequential (n=1736) and simultaneous (n=1638) conditions received tailored motivational feedback to change unhealthy behaviors one at a time (sequential) or all at the same time (simultaneous). Mixed model analyses were performed as primary analyses; regression analyses were done as sensitivity analyses. An overall risk score was used as outcome measure, then effects on the 5 individual lifestyle behaviors were assessed and a process evaluation was performed regarding exposure to and appreciation of the intervention.

**Results:**

Both tailoring strategies were associated with small self-reported behavioral changes. The sequential condition had the most significant effects compared to the control condition after 12 months (T1, effect size=0.28). After 24 months (T2), the simultaneous condition was most effective (effect size=0.18). All 5 individual lifestyle behaviors changed over time, but few effects differed significantly between the conditions. At both follow-ups, the sequential condition had significant changes in smoking abstinence compared to the simultaneous condition (T1 effect size=0.31; T2 effect size=0.41). The sequential condition was more effective in decreasing alcohol consumption than the control condition at 24 months (effect size=0.27). Change was predicted by the amount of exposure to the intervention (total visiting time: beta=–.06; *P*=.01; total number of visits: beta=–.11; *P*<.001). Both interventions were appreciated well by respondents without significant differences between conditions.

**Conclusions:**

Although evidence was found for the effectiveness of both programs, no simple conclusive finding could be drawn about which intervention mode was more effective. The best kind of intervention may depend on the behavior that is targeted or on personal preferences and motivation. Further research is needed to identify moderators of intervention effectiveness. The results need to be interpreted in view of the high and selective dropout rates, multiple comparisons, and modest effect sizes. However, a large number of people were reached at low cost and behavioral change was achieved after 2 years.

**Trial Registration:**

Nederlands Trial Register: NTR 2168; http://www.trialregister.nl/trialreg/admin/rctview.asp?TC=2168 (Archived by WebCite at http://www.webcitation.org/6MbUqttYB).

## Introduction

Since the development of the Internet, several types of Web-based interventions have been offered to the population to modify unhealthy lifestyle behaviors. An unhealthy lifestyle can be described as one that is not compliant with the guidelines for different prominent health risk behaviors, such as being insufficiently active, eating insufficient fruit and vegetables, drinking too much alcohol, and using tobacco [1,2]. Unhealthy lifestyle habits are among the main causes of mortality and morbidity [3]. Noncommunicable chronic diseases, such as heart diseases, cancer, diabetes, and chronic respiratory diseases [1], are associated with a limited number of common modifiable health behaviors [4].

Given the high prevalence of unhealthy lifestyle habits [5-8], it is reasonable to offer interventions that can be disseminated among large numbers of people at low cost [9]. Because individuals with multiple unhealthy lifestyle behaviors are at the greatest risk of developing chronic diseases leading to increased health care costs [10], Web-based interventions with a focus on different lifestyle behaviors and integrated within one intervention seem to be an appropriate choice. To increase changes in health behavior, tailored interventions using a computerized expert system to select the best-fitting messages to generate personal relevant feedback messages have been developed [11]. Although inconsistent findings are reported [12,13], many Web-based computer-tailored interventions have proven to be an effective tool for improving health-related behaviors (eg, [14-16]). Different interventions based on the I-Change model have shown positive results [17-19]. As shown by Webb et al [20], the use of theory results in larger effect sizes. Moreover, Web-based computer-tailored interventions that contain relevant and attractive information adapted to the respondents’ individual characteristics and needs have proven to be cost-effective (eg, [21,22]) and have been evaluated more positively than general information [8].

It has been suggested that interventions that focus on multiple behaviors have a greater impact on public health than single-behavior interventions [8,23]. However, such interventions are more extensive and, thus, require more engagement, time, and effort from the respondent. There is limited and inconsistent evidence about how best to accomplish multiple behavior change when using Web-based computer-tailored interventions. One strategy is to intervene in a single behavior at a time (sequential approach); another approach is to intervene in all health risk behaviors at the same time (simultaneous approach) [8,24-30]. In earlier studies, no consistent findings were reported regarding the most effective strategy. For example, King et al [25] and Hyman et al [24] reported that their simultaneous intervention mode was superior to their sequential intervention mode. The first aimed at changes in diet and physical activity; the latter aimed at smoking cessation and improvements in diet and physical activity. A study by Vandelanotte et al [30] aimed at lowering fat intake and increasing physical activity found no differences between the sequential and the simultaneous condition.

Focusing on different behaviors sequentially at different points in time is associated with less cognitive effort during the individual visits; however, respondents may experience lower levels of autonomy because of the limited choices during the individual visits [31,32], and repeated participation is necessary to receive information about multiple behaviors. Intervening in all behaviors simultaneously has the advantage that respondents receive all relevant information during the first visit; however, such a program may lead to ego depletion by overwhelming respondents with too much information [33,34] which may lead to a more negative feeling regarding the intervention and immediate dropout. In previous research, dose-response relationships have been found between exposure to an intervention (number and duration of exposures) and behavior change outcomes [35,36]. This may imply that repeated participation and thus repeated exposure to the Web-based program can be beneficial for realizing substantial behavioral change [37]. Appreciation of the intervention may lead to increased use, which, in turn, may improve intervention effectiveness [18,38,39]. Thus, both delivery modes have potential advantages and disadvantages that may influence their effectiveness, use, and the appreciation of the different types of interventions. Current evidence regarding the effectiveness of sequential and simultaneous delivery modes is inconsistent and none of these studies to date have tested the 2 delivery modes within an intervention targeting 5 different health behaviors.

In summary, randomized controlled trials assessing the effects, especially the long-term effectiveness (after more than 1 year) [40], of computer-tailored multiple-lifestyle interventions using different strategies (sequential vs simultaneous) among adults are scarce. Therefore, the main aim of the current study was to assess whether a multisession, Web-based, tailored lifestyle intervention was effective in enhancing multiple lifestyle behaviors (ie, physical activity, vegetable consumption, fruit consumption, alcohol intake, and smoking) in the long term. First, potential differences in effects of lifestyle change in general were assessed between the sequential and simultaneous delivery mode and a control condition at 12-month and 24-month follow-up. Second, we evaluated whether there were differences between the 3 groups with regard to effects on adherence to individual guidelines for each of the 5 behaviors being assessed. As a tertiary objective, a process evaluation was executed by studying the influence of intervention exposure on effectiveness and by summarizing the appreciation of the intervention.

## Methods

### Overview

A detailed description of the study protocol has been published elsewhere [27]; therefore, a summary of study methodology and procedures is provided subsequently.

### Participants, Procedure, and Study Design

We conducted a randomized controlled trial (Dutch Trial Register NTR2168), involving 2 experimental conditions and a control condition. The Web-based intervention, focusing on unhealthy lifestyle behaviors in the general population, was conducted in the Netherlands from November 2009 to July 2012. Follow-up measurements took place 12 (T1) and 24 months (T2) after the first intervention visit. In the first instance, adult participants were recruited via 4 Dutch Regional Health Authorities that had conducted the quadrennial Adult Health Monitor 2009 of inhabitants of the provinces of North-Brabant and Zeeland (N=96,388). This monitoring tool is used to assess general health (eg, physical and mental health) and cover health-related topics (eg, social and physical environment) among representative samples of the Dutch population [41]. Of the 41,155 (42.70%) respondents who completed the Monitor, 24,215 (58.84%) filled out the written version and 16,940 (41.16%) filled out the online version (see also [42]). Our intervention was partly integrated into the online version of the Monitor. At the end of this Web-based questionnaire, participants were invited to take part in our intervention study. They had to give informed consent and provide their email address for participation and the handling of their data. Three weeks later, the study sample received an email containing an invitation and a link to the intervention website. After 1 month, a reminder email was sent to the individuals in the sample who had not responded to the first invitation. The website was also open to the general public, so it was also possible to register directly on the website without having to complete the Monitor. Randomization to 1 of the 3 study groups took place by means of a computer software randomization system. No block or cluster randomization was applied; the randomization was done at the individual level. The following inclusion criteria were established for this study: being between ages 18 and 65 years, having a computer with Internet access, having basic Internet literacy, and having a valid email address. The study was approved by the Medical Ethics Committee of Maastricht University and the University Hospital Maastricht (MEC 09-3-016/NL27235.068.09).

### Intervention Program

#### Overview

The intervention program, called myHealthyBehavior (Dutch: *mijnGezondGedrag*), was a Web-based computer-tailored program targeting adults. The main aim of the intervention was to motivate participants to enhance 5 health behaviors. The theoretical framework for the development of the intervention was the I-Change model [43].

#### The Health Risk Appraisal

In the first part of the intervention, all groups received a health risk appraisal (HRA) in which the 5 behaviors were placed in the context of the following Dutch public health guidelines for each of the different behaviors: being moderately physically active for 30 minutes on at least 5 days a week [44], eating 200 g of vegetables per day [45], eating 2 pieces of fruit per day [45], not drinking more than 1 (women) or 2 (men) glasses of alcohol a day [45], and not smoking [46]. Figures with pictures of traffic lights were presented for every behavior to indicate whether respondents met (green), almost met (orange), or did not meet (red) the guidelines.

#### Sequential and Simultaneous Delivery Modes

**Figure 1:**
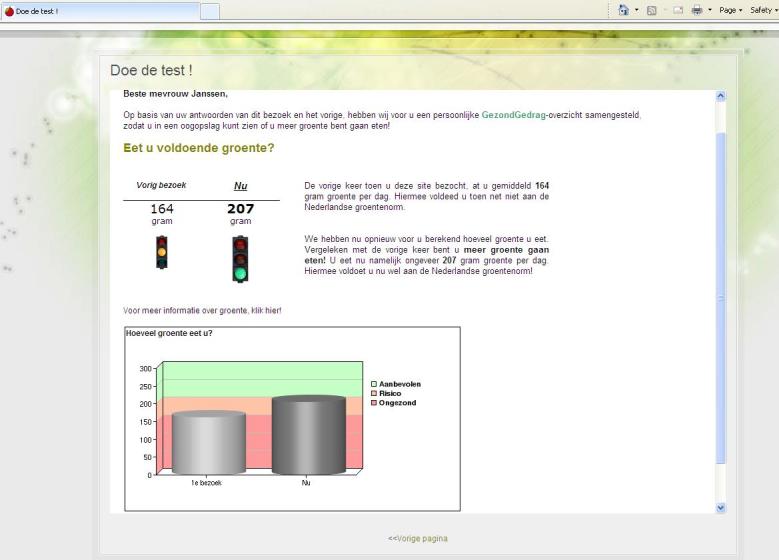
Screenshot of the intervention website showing the vegetable part of the health risk appraisal. The traffic lights show that the respondent did not comply with the vegetable guideline at her first visit (orange traffic light), but that she complied with the guideline at her second visit (green traffic light). The graph shows the scores (amount of vegetables) at all visits.

The second part of the intervention was only available for the 2 experimental conditions: (1) the sequential condition and (2) the simultaneous condition. In this part, self-assessed questionnaires were used to measure the psychosocial concepts of the I-Change model [43]. The feedback messages were based on these questionnaires and were revealed on the respondents’ computer screens immediately after the completion of the surveys. Respondents who did not comply with at least 1 guideline were invited to change the behavior which they had not complied with. They were invited to complete additional questions regarding motivational constructs related to a chosen behavior (sequential condition) or all relevant behaviors (simultaneous condition) within 1 or more modules of the intervention, respectively. Motivational feedback was given that was related to the relevant behaviors. Feedback on each person’s perception of the pros and cons of the health behavior (attitudinal feedback) was given as the first step of the program, followed by feedback on perceived social influences as the second step. For example, information was given on how the social environment could help the respondent to live healthily. In the third step, the concept of preparatory planning was addressed. Feedback was provided on how to prepare for behavior change, for example, by planning to be physically active at fixed times, adding the plans to an agenda, trying new/different kinds of sports, having enough vegetables in stock at home, eating fruit or vegetables as a snack, and (for smokers) planning a quit date. In the final step, the focus was placed on how to cope with difficult situations to also increase self-efficacy. Several tailoring strategies were used in the feedback messages; for example, respondents were addressed by their name, normative and ipsative feedback were given (ie, during revisits current scores were compared to scores of previous visits; see Figure 1), graphs and bar charts were included, and a personal tone and empathy was applied.

It was a multisession program, in which respondents were encouraged to revisit the website on an unlimited basis. After 1 year, respondents in the sequential condition had the opportunity to choose a second module and to receive feedback on another lifestyle behavior. Respondents in the simultaneous condition received feedback on all behaviors for which they did not meet the guideline simultaneously at baseline and after 12 months. When visiting the website in the meantime, the sequential group received feedback on the chosen behavior at baseline or at 12-month follow-up, respectively; the simultaneous group received feedback on all unhealthy behaviors they reported at that moment. After 24 months, an invitation to complete the last follow-up questionnaire was sent to all respondents, followed by 2 reminders to increase the response rate.

### Questionnaires

#### Demographic Information

We assessed 8 demographic variables: age, gender (male=1; female=2), educational level (low=1, no education or primary education; medium=2, secondary education; high=3, tertiary education), net household income (euros per month), employment situation, marital status, number of persons in household, and native country (The Netherlands=1; other country=2).

#### Health Status

We assessed different kinds of diseases, such as cardiovascular diseases, diabetes, cancer, and high blood pressure. The 12-item short form (SF-12) Health Survey [47,48] was used to assess quality of life. The Kessler Psychological Distress Scale (K10) [49] was included to assess symptoms of depression and anxiety. The items regarding height and weight were used to estimate the body mass index (BMI).

#### Lifestyle Behavior

Physical activity was measured by the short questionnaire to assess health-enhancing physical activity (SQUASH) [41,50], and guideline adherence was assessed using comparable procedures developed by Ainsworth et al [51]. Fruit consumption was measured using a 4-item food frequency questionnaire (FFQ) assessing weekly fruit and fruit juice intake [41]. Vegetable consumption was measured using a 4-item FFQ assessing the weekly consumption of boiled or baked vegetables, as well as salads or raw vegetables [41]. Alcohol intake was measured by the 5-item Dutch Quantity-Frequency-Variability (QFV) questionnaire [52].

Current smoking behavior was assessed by asking participants if they smoked, what they smoked (cigarettes, cigars, or pipe tobacco), and how much they smoked per day (cigarettes) or per week (cigars or pipe tobacco) [41]. The answers were converted into an overall score for tobacco consumption (expressed in number of cigarettes): 1 cigar=4 cigarettes and 1 package of pipe tobacco (50 g)=50 cigarettes [53].

#### Social Cognitive Variables

Based on earlier studies [8,15], the following social cognitive variables were assessed regarding 1 or more of the 5 different lifestyle behaviors (dependent on the study condition). These variables were used to compose the personalized advice.

Intention was assessed by 1 item per behavior using an extended version of the stage of change concept [54,55]. For example, for the question “Do you intend to be physically active for 30 minutes on at least 5 days a week?” answers included no, I don’t intend to do so (=1); I never thought about it (=2); I thought about it, but I don’t know yet (=3); yes, but not within the next 5 years (=4); yes, within 1 to 5 years (=5); yes, within 6 to 12 months (=6); yes, within 3 to 6 months (=7); yes, within 1 to 3 months (=8); yes, within a month (=9); yes, and I’m already doing so (=10).

Attitude was assessed by 3 pros per behavior, such as “regular physical activity is good for my health” with responses ranging from totally disagree (=1) to totally agree (=5); and by 3 cons, such as “regular physical activity costs a lot of time” from totally disagree (=1) to totally agree (=5).

Social influence was assessed by social norms (1 item), social modeling (1 item) and social support (1 item), such as “According to the people in my direct environment...” with answers ranging from I certainly should smoke (=1) to I certainly should not smoke (=5); “How many people in your direct environment smoke?” with answers ranging from nobody (=1) to everybody (=5); and “People from my direct environment support me not to smoke” with answers ranging from yes, they support me a lot (=4) to no, they do not support me at all (=1).

Preparatory plans were assessed by 3 items per behavior, such as “I intend to allow time for physical activity” with answers ranging from no, definitely not (=1) to yes, definitely (=5).

Self-efficacy was assessed by 6 items per behavior regarding difficult social, emotional, and routine situations, such as “I am able to meet the alcohol guideline...when I am at a party;...when I feel stressed or nervous;...during a meal,” or “I am able to eat sufficient vegetables when I have other delicious food at home” with answers ranging from no, definitely not (=1) to yes, definitely (=5).

Coping plans were assessed by 6 items per behavior, such as “I have made a plan to drink no more than 2 glasses of alcohol when I feel stressed or nervous” with answers ranging from totally disagree (=1) to totally agree (=5).

### Process Evaluation

To report user statistics, we recorded the number of log-ins to the tailored intervention per respondent and the time respondents spent on the tailored intervention during each visit. At baseline, after 12 months, and after 24 months, respondents evaluated the HRA on a scale from very bad (=1) to excellent (=10). At 24-month follow-up, all respondents were invited to complete 4 more questions measuring appreciation of the program by assessing the user-friendliness of the website and satisfaction with the layout, the HRA in general, and the use of traffic lights in particular. Additionally, a subsample completed a separate questionnaire, including items evaluating the website (6 items), the HRA (13 items), and the personalized advice (15 items).

### Power Analyses

Linear mixed model analysis (lifestyle factor is the outcome variable) was the main analysis and power analysis suggested that a total sample of 1182 respondents was needed (correcting for possible attrition) based on *P*=.05, a power of 80%, a 2-sided test, and an effect size (ES) of 0.20. For the logistic analyses, a sample of 882 respondents was needed.

### Statistical Analyses

The data were analyzed using SPSS software version 20 (IBM Corp, Armonk, NY, USA). To examine whether the randomization had been successful, the 3 groups were compared in terms of demographics, health status, and lifestyle behavior; ANOVAs were executed for continuous variables and chi-square tests for discrete variables. In the case of significant differences, variables were included as covariates (ie, potential confounders) in subsequent analyses.

Descriptive statistics were used to describe the characteristics of the study sample and the dropout rate within the groups. Dropout analyses, including a comparison between respondents lost to follow-up and T0-T1 completers (ie, respondents who completed the baseline and the 12-month follow-up measurement) and T0-T2 completers (ie, respondents who completed the baseline and the 24-month follow-up measurement), respectively, and fully complete cases were done using ANOVAs and chi-square tests.

We calculated a risk factor score by summing up all risky/unhealthy behaviors defined by the guideline status; the value for the risk factor score could range from zero (adhering to all guidelines) to 5 (adhering to no guideline). The 3 study groups were compared in terms of their lifestyle behavior at the follow-up measurements. First, ES were calculated (Cohen’s *d*). Those ES below 0.30 were considered small, whereas those between 0.30 and 0.80 were considered medium, and those larger than 0.80 were regarded as large [56]. Second, repeated measures analyses using the top-down procedure were conducted to study changes during the study period (time) and differences in changes between the study groups (time × condition). Linear mixed model analyses were used for the analyses with the risk factor score as outcome measure. Logistic mixed model analyses were used with the guidelines status for the 5 lifestyle behaviors as outcome measures. These kinds of analyses allow for inclusion of all cases (despite missing values of the outcome variable), and are valid in case the missing values satisfy the missingness at random assumption [57].

For the sensitivity analyses, differences in effect between the groups were explored by means of linear and logistic regression analyses by using the top-down procedure. The dependent variables were the risk factor score and complying with the physical activity guideline (yes=1; no=0), complying with the vegetable guideline (yes=1; no=0), complying with the fruit guideline (yes=1; no=0), complying with the alcohol guideline (yes=1; no=0), and complying with the smoking guideline (yes=1; no=0). All analyses were done for both the 12-month and 24-month follow-up measurement. To increase power and external validity, these regression analyses were first performed for T0-T1 completers and T0-T2 completers, respectively. Next, these analyses were also performed based on fully complete cases (ie, respondents who completed both follow-up measurements). The results of the sensitivity analyses are outlined in Multimedia Appendices 1-2.

Among the experimental conditions, linear regression analyses were performed to study the predictive value of the total visiting time of the intervention and the total number of visits during the study period on the risk factor score after 24 months. Descriptive statistics were used to describe the evaluation/appreciation of the intervention at different time points.

Tests were performed at alpha=.05 for the intervention factor and alpha=.10 for covariates [58].

## Results

### Participation and Sample Characteristics

**Figure 2:**
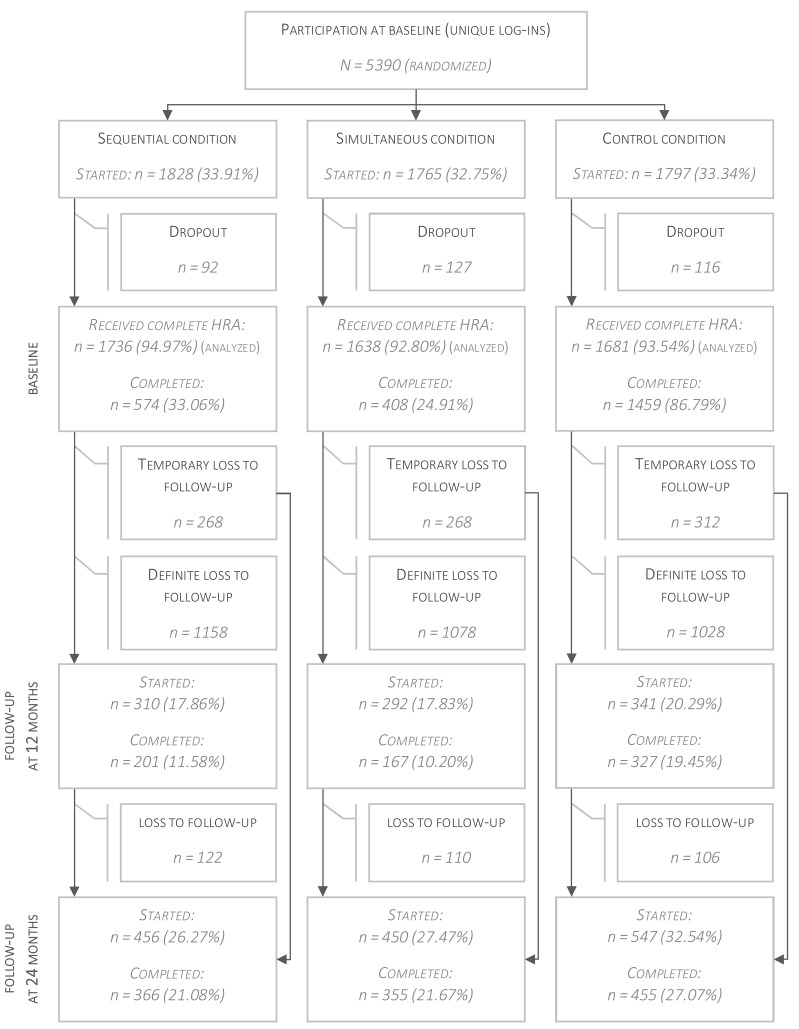
Flowchart of study participants. Completed: respondents who adhered to all study protocols; temporary loss to follow-up: respondents who did not complete the follow-up at 12 months, but did at 24 months; started: respondents who logged into intervention, but did not complete assessment.

Figure 2 shows the flow of the respondents from enrollment in the study to allocation to the 3 different conditions, and revisits after 12 and 24 months. In total, 5055 respondents were included in the analyses of this study, of which 4833 (95.61%) were participants of the Adult Health Monitor. A description of the study sample is shown in Table 1. We found some baseline differences between the 3 study groups. The age of respondents in the control condition was significantly higher when compared to the age in the sequential condition (*P*=.03). More respondents in the experimental conditions suffered from heart attacks (*P*=.01), but fewer people reported high blood pressure (*P*=.002). Compliance rates regarding vegetable intake were higher in the simultaneous group than in the control condition (*P*=.07) although this did not reach statistical significance; and respondents in the sequential condition reported smoking more cigarettes than respondents in the control condition (*P*=.04).

**Table 1:** Demographics, health status, and lifestyle behavior of the study sample at baseline.

Variable			Total N=5055	Sequential n=1736	Simultaneous n=1638	Control n=1681	*P*
**Demographics**							
	Age (range 19-65 years), mean (SD)		44.15 (12.67)	43.70 (12.62)	43.94 (12.57)	44.82 (12.81)	.03^a^
	**Gender, n (%)**						
		Male	2661 (52.64)	893 (51.44)	878 (53.60)	890 (52.94)	
		Female	2394 (47.36)	843 (48.56)	760 (46.40)	791 (47.06)	
	**Education (n=4961), n (%)**						
		Low	515 (10.38)	167 (9.88)	174 (10.84)	174 (10.45)	
		Medium	2334 (47.05)	809 (47.84)	731 (45.55)	794 (47.69)	
		High	2112 (42.57)	715 (42.28)	700 (43.61)	697 (41.86)	
	**Income (€/month; n=4970), n (%)**						
		<1750	1165 (23.44)	399 (23.51)	373 (23.23)	393 (23.58)	
		1751-3050	1688 (33.96)	573 (33.77)	543 (33.81)	572 (34.31)	
		>3051	1397 (28.11)	465 (27.40)	466 (29.02)	466 (27.95)	
		Not reported	720 (14.49)	260 (15.32)	224 (13.95)	236 (14.16)	
	**Employment situation (n=4970), n (%)**						
		Job (paid employment)	3788 (76.22)	1290 (75.97)	1240 (77.26)	1258 (75.46)	
		No job	1182 (23.78)	408 (24.03)	365 (22.74)	409 (24.54)	
	**Marital status (n=4957), n (%)**						
		Relationship	3775 (76.15)	1292 (76.27)	1215 (75.94)	1268 (76.25)	
		Single	1182 (23.85)	402 (23.73)	385 (24.06)	395 (23.75)	
	Persons in household (n=4980), mean (SD)		2.9 (1.42)	2.93 (1.47)	2.89 (1.37)	2.88 (1.42)	
	**Native country (n=4973), n (%)**						
		The Netherlands	4727 (95.05)	1613 (94.99)	1531 (95.27)	1583 (94.90)	
		Other	246 (4.95)	85 (5.01)	76 (4.73)	85 (5.10)	
**Health status**							
	BMI (range 13.82-58.11; n=5012), mean (SD)		25.20 (4.03)	25.26 (4.01)	25.15 (3.99)	25.17 (4.09)	
	Quality of life (range 15-48; n=4925), mean (SD)		40.08 (5.24)	40.02 (5.28)	40.19 (5.09)	40.03 (5.34)	
	Psychological distress (range 12-50; n=4944), mean (SD)		44.77 (5.74)	44.69 (5.83)	44.86 (5.62)	44.76 (5.76)	
	**Disease (n=4950), n (%)**						
		Diabetes	144 (2.91)	48 (2.84)	44 (2.74)	52 (3.14)	
		Brain hemorrhage, TIA	19 (0.38)	4 (0.24)	8 (0.50)	7 (0.42)	
		Heart attack	39 (0.79)	22 (1.30)	11 (0.68)	6 (0.36)	.003^a^; .001^b^; .08^c^
		Other serious heart disease	61 (1.23)	21 (1.24)	21 (1.31)	19 (1.15)	
		Cancer	67 (1.35)	24 (1.42)	19 (1.18)	24 (1.45)	
		High blood pressure	618 (12.4)	200 (11.79)	174 (10.83)	244 (14.69)	.01^a^; .001^b^
		Asthma, COPD	333 (6.73)	109 (6.45)	110 (6.84)	114 (6.88)	
		One or more diseases	1048 (21.17)	348 (20.58)	324 (20.16)	376 (22.76)	
**Lifestyle behavior**							
	**Number of risk factors (n=4965), n (%)**						
		0	547 (11.02)	196 (11.48)	178 (11.05)	173 (10.51)	
		1	1413 (28.46)	476 (27.87)	458 (28.43)	479 (29.10)	
		2	1779 (35.83)	589 (34.48)	576 (35.75)	614 (37.30)	
		3	935 (18.83)	334 (19.56)	298 (18.50)	303 (18.41)	
		4	262 (5.28)	102 (5.97)	90 (5.59)	70 (4.25)	
		5	29 (0.58)	11 (0.64)	11 (0.68)	7 (0.43)	
	Number of risk factors, mean (SD)		1.81 (1.07)	1.83 (1.10)	1.81 (1.08)	1.78 (1.03)	
	**Physical activity (n=5053)**						
		Minutes per day, mean (SD)	159.32 (160.84)	158.78 (160.12)	155.57 (159.05)	163.53 (163.30)	
		Noncompliance, n (%)	651 (12.88)	235 (13.54)	220 (13.44)	196 (11.67)	
	**Vegetable consumption (n=5018)**						
		Number of grams, mean (SD)	172.49 (85.07)	171.19 (83.61)	173.97 (82.94)	172.40 (88.58)	
		Noncompliance, n (%)	3419 (68.13)	1166 (67.63)	1085 (66.56)	1168 (70.19)	.03^b^
	**Fruit consumption (n=5019)**						
		Pieces of fruit, mean (SD)	1.94 (1.29)	1.94 (1.27)	1.93 (1.33)	1.96 (1.28)	
		Noncompliance, n (%)	2710 (53.99)	946 (54.90)	892 (54.86)	872 (52.22)	
	**Alcohol intake (n=5034)**						
		Number of drinks, mean (SD)	1.26 (1.57)	1.22 (1.46)	1.31 (1.64)	1.25 (1.61)	
		Noncompliance, n (%)	1405 (27.91)	488 (28.19)	453 (27.77)	464 (27.75)	
	**Smoking (n=5055)**						
		Number of cigarettes, mean (SD)	2.25 (6.47)	2.48 (7.34)	2.33 (6.05)	1.94 (5.86)	.09^a^
		Noncompliance, n (%)	897 (17.74)	321 (18.49)	302 (18.44)	274 (16.30)	

^a^Sequential vs control.

^b^Simultaneous vs control.

^c^Sequential vs simultaneous.

### Dropout Analyses

Respondents followed up and respondents lost to follow-up differed on a number of variables. Dropout was associated with some demographic factors (eg, a younger age), a better health status as indicated by fewer diseases and lower BMI, but an unhealthier lifestyle. More detailed information can be found in Multimedia Appendix 3.

### Intervention Effects

**Figure 3:**
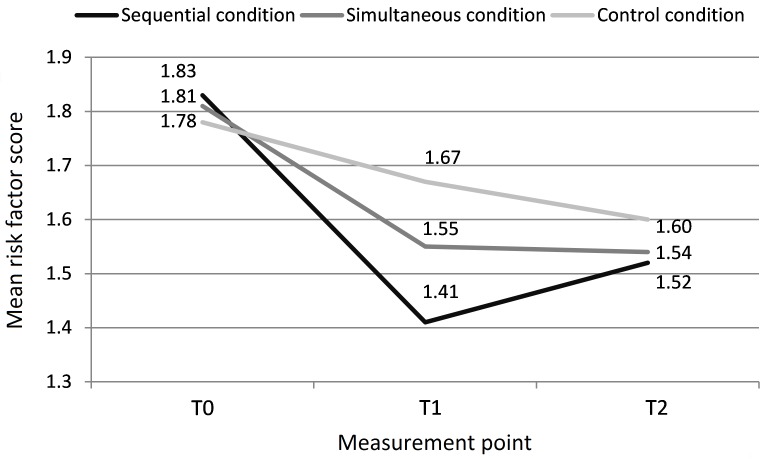
Mean number of risk factors among the different conditions at baseline (T0) and at 12-month (T1) and 24-month (T2) follow-ups.

We assessed the effects of the intervention on the overall lifestyle risk factor. The higher the score on the risk factor, the more a respondent did not comply with the Dutch guidelines concerning the lifestyle behaviors. The results of the linear mixed model analyses show that the risk factor score changed favorably and significantly over time (Figure 3) and that there is a statistically significant difference between the experimental conditions and the control condition (Table 2). After 12 months, the sequential condition was significantly more effective in reducing the risk factor score compared to the control condition. A similar but not statistically significant effect (*P*=.08) was found for the simultaneous condition compared to the control condition. After 24 months, only the simultaneous condition showed a statistically significant effect compared to the control condition, revealing a significantly lowered risk score for participants in the simultaneous condition. On both follow-up measurements, there were no statistically significant differences regarding the risk factor score between the sequential and the simultaneous condition.

The sensitivity analyses showed similar results (see Multimedia Appendices 1 and 2), except for 2 differences: (1) among the T0-T1 completers, both simultaneous and sequential interventions were effective in reducing the risk factor score after 12 months compared to the control condition, and (2) among fully complete cases, both simultaneous and sequential interventions revealed significant effects in reducing the risk factor score in comparison to the control condition after 24 months.

**Table 2:** Results of linear mixed model analyses (top-down procedure)^a^ with the risk factor score after 12 and 24 months as outcome measure.

Condition and time		*P*	Effect size
**Type III tests**			
	Condition × time	.04	—
	Time	<.001	—
**After 12 months (T1)**			
	Sequential vs control	.008	0.28
	Simultaneous vs control	.08	0.19
	Sequential vs simultaneous	.39	0.10
**After 24 months (T2)**			
	Sequential vs control	.13	0.14
	Simultaneous vs control	.048	0.18
	Sequential vs simultaneous	.68	0.04

^a^All variables regarding demographics, health status, and lifestyle behavior were included in the most extensive model.

### Differences in Lifestyle Behaviors

We conducted in-depth analysis to assess the effects of the interventions for each lifestyle behavior separately. Table 3 summarizes the changes in the 5 different health risk behaviors according to the guideline status. The logistic mixed model analyses showed that all 5 lifestyle behaviors changed over time, but only a few effects differed significantly between the conditions. At both follow-up measurements, the sequential condition was found to result in significant changes in smoking abstinence in comparison to the simultaneous condition. After 24 months, the sequential condition had greater effect than the control condition, although this did not meet statistical significance (*P*=.06). After 24 months, the sequential condition was more effective in decreasing alcohol consumption than the control condition. The differences between both conditions, although not statistically significant (*P*<.10), indicates that the simultaneous condition might have been more effective in increasing the amount of physical activity compared to the sequential condition after 12 months, and in increasing fruit intake (after 12 and 24 months) compared to the control condition.

However, when performing the sensitivity analyses, no consistent pattern could be found. Differences in statistical significance were found, especially in comparisons between groups with regard to fruit and vegetable intake, alcohol consumption, and smoking. More information can be found in Multimedia Appendices 1 and 2.

**Table 3:** Results of the logistic mixed model analyses (top-down procedure)^a^ with the specific lifestyle behavior (guideline status) after 12 and 24 months as outcome measure.

Condition and time		Outcome measure									
		Physical activity		Vegetable		Fruit		Alcohol		Smoking	
		*P*	ES	*P*	ES	*P*	ES	*P*	ES	*P*	ES
**Fixed effects**											
	Condition × time	.30	—	.65	—	.21	—	.27	—	.045	—
	Time	.04	—	.046	—	.13	—	<.001	—	<.001	—
**After 12 months (T1)**											
	Sequential vs control	.23	0.28	.26	0.20	.61	0.08	.66	0.07	.42	0.13
	Simultaneous vs control	.52	0.17	.41	0.15	.06	0.29	.24	0.19	.23	0.18
	Sequential vs simultaneous	.07	0.45	.78	0.05	.18	0.21	.49	0.12	.048	0.31
**After 24 months (T2)**											
	Sequential vs control	.43	0.18	.62	0.07	.30	0.14	.048	0.27	.06	0.27
	Simultaneous vs control	.48	0.16	.66	0.07	.07	0.24	.20	0.17	.26	0.15
	Sequential vs simultaneous	.94	0.02	.37	0.14	.46	0.10	.49	0.10	.004	0.41

^a^All variables regarding demographics, health status, and lifestyle behavior were included in the most extensive model.

### Process Evaluation

#### Exposure to the Intervention

When comparing the total visiting time and the total number of visits in the intervention during the 24 months, statistically significant differences between the 3 groups were found. On average, respondents in the sequential condition visited the intervention for 31 (SD 40) minutes, respondents of the simultaneous condition stayed on the website for 28 (SD 36) minutes, and respondents in the control condition visited the website for 16 (SD 21) minutes (*F*_2,1124_=23.78; sequential vs control: *P*<.001; simultaneous vs control: *P*<.001; sequential vs simultaneous: *P*=.31). The mean number of visits in the sequential condition was 2.04 (SD 1.35), in the simultaneous condition this was 2.01 (SD 1.45), and in the control condition this was 1.85 (SD 0.93; *F*_2,1124_=2.84; sequential vs control: *P*=.75; simultaneous vs control: *P*=.16; sequential vs simultaneous: *P*=.91). The regression analyses conducted among respondents in the sequential and the simultaneous conditions only showed that the risk factor after 24 months was predicted by the total visiting time (beta=–.06; *P*=.01) and the total number of visits during the study period (beta=–.11; *P*<.001). Longer visits and a greater number of visits predicted more favorable risk factor changes.

#### Appreciation of the Intervention

The HRA was evaluated at all 3 measurement points. At baseline, the HRA score was evaluated as mean 7.2 (SD 1.3; n=2441), after 12 months the HRA was evaluated as mean 7.3 (SD 1.1; n=368), and after 24 months it was evaluated as mean 7.6 (SD 1.0; n=1176). No differences existed between the experimental conditions; however, at baseline, the HRA was more positively evaluated by the sequential condition (mean 7.4, SD 1.1; n=574) and the simultaneous condition (mean 7.3, SD 1.4; n=408) than the control condition respondents (mean 7.1, SD 1.3; n=1459; *F*_2,2438_=16.48; *P*<.001). Of those respondents who completed the last follow-up measurement, 84.43% (998/1182) stated that the HRA gave a good overview of their lifestyle, 77.58% (917/1182) liked the use of traffic lights in the HRA, 72.33% (852/1178) liked the layout, and 76.74% (904/1178) experienced website use as user friendly.

Additional evaluations of the intervention, filled out by 305 respondents, revealed no statistically significant differences between the conditions with regard to the website and the HRA. The evaluation of the tailored advice among respondents of the sequential and simultaneous condition revealed that the respondents evaluated the personalized advice as relevant (75.4%, 86/114), credible (76.5%, 88/115), informative (70.4%, 81/155), well arranged (84.3%, 97/115), clear (85.1%, 97/114), interesting (71.3%, 82/115), and with an attractive layout (70.0%, 77/115). The personalized advice was evaluated as mean 6.9 (SD 1.3), with no statistically significant differences between conditions. Almost 80% (79.8%, 91/114) reported having read all parts of the advice, 40.9% (47/115) reported that they wanted to live a healthier life because of the received advice, and 16.5% (19/115) found the advice too long. One statistically significant difference was found: respondents in the sequential condition indicated more often that they missed information in the advice than respondents in the simultaneous condition (*t*_111.49_=2.01; *P*=.047). Some suggestions were given for improving the intervention; for example, attention should be paid to personal circumstances by asking more specific questions about reasons for not eating sufficient vegetables or for being insufficiently physically active, and the advice could be made more personal by giving more concrete examples.

## Discussion

### Effectiveness of the Intervention

The primary aim of this study was to evaluate the effectiveness of 2 computer-tailored Web-based interventions compared to a control condition with regard to lifestyle improvement. Overall, the computer-tailored intervention for multiple health behaviors resulted in favorable lifestyle changes. Compared to the control condition, the sequential delivery mode was found to be most effective after 12 months, whereas the simultaneous delivery mode was most effective after 24 months. The sensitivity analyses yielded comparable results and suggested slightly stronger effects for both delivery modes, which may be because those who were motivated to fill out more postmeasurements were also those who were more motivated to change. The effect sizes were small which is common for computer-tailored interventions, but they can still result in a large public health impact when widely implemented [14,59]. For instance, for smoking cessation a small ES is considered clinically significant [60], which may also be relevant for other behaviors addressed in our study. Moreover, our control condition received a minimal intervention (ie, the tailored HRA). Personalized information regarding one’s lifestyle behavior may be sufficient to facilitate change and improve lifestyle behaviors [61]. Further studies are needed that compare a sequential and simultaneous condition for a group that receives general information or no information at all.

Regarding the overall lifestyle behavior changes, no differences were found between the sequential and the simultaneous condition. This is in-line with the findings Vandelanotte et al [30] reported in their study aimed at lowering fat intake and increasing physical activity. We assume that only those respondents with the highest motivational level to change remained in our study at follow-up. Therefore, it might be that no differential effects were found between the sequential and simultaneous condition.

When analyzing the separate behaviors, the largest changes were found for smoking cessation, followed by lower alcohol intake and increased fruit consumption. However, these findings were only partly replicated in the sensitivity analyses. The results of the sensitivity analyses supposed that the effect on the overall risk factor change can primarily be ascribed to changes in fruit consumption, vegetable consumption, alcohol intake, and probably in tobacco use. Hence, no firm conclusion can be drawn with regard to the differential effects on separate lifestyle behaviors. Further research is needed to investigate whether the optimum tailoring strategy (ie, a sequential, simultaneous, or even single-behavior approach) may depend on the behavior(s) being targeted.

### Dropout Rates

An important limitation of our study is the high dropout rates. Mixed model analyses were performed to increase internal and external validity; however, it is not unlikely that informative dropout occurred (ie, the dropout process depends on the unobserved measurements) [57,62]. This would be a violation of the missing at randomness assumption underlying the mixed model analyses. The results of the sensitivity analyses, using regression techniques, might not be generalizable to the general population, but only to those people who remain participating in such a study over a longer period of time. As shown in our attrition analyses study [63], younger people and those with an unhealthier lifestyle were more likely to drop out.

We made use of a low-intensity implementation in the current study (recruitment via Regional Health Authorities, sending emails without face-to-face or telephone contact for the entire study). This implementation strategy may have led to relatively high attrition [64,65]. Yet, our dropout levels are comparable with those of other website-delivered studies with similar protocols [66-68] and some participants indicated (via email) that they did not revisit the intervention because they had complied with all 5 guidelines and found that they did not need more information. Furthermore, factors of the intervention itself may have caused dropout, such as technical issues or problems of navigating through the intervention website. As hypothesized in the Model of Internet Interventions, website characteristics, such as appearance, behavioral prescriptions, burdens, content, delivery, message, participation, and assessment, may influence website use and effectiveness [69].

### Exposure Rates

There is growing evidence that the level of engagement in eHealth plays an important role in explaining the use and effectiveness of Web-based interventions. It is challenging to develop eHealth interventions focused on multiple health behaviors that engage participants sufficiently to revisit the website on more than 1 occasion. As our results suggest, higher usage is related to higher effects, which has also been observed in other studies [70,71]. With regard to the time respondents spent on the website, it is striking that many really short visits (<20 seconds) were recorded. This is in-line with the findings of Brouwer et al [72] who reported that more than half of their visitors left the website within 30 seconds. Internet interventions have the disadvantage that clicking off the website is easy. Respondents may have opened the link without knowing what they were opening (in our case, not reading the email invitations to visit our intervention) resulting in loss of interest after opening the website. On the other hand, some quite long visits were recorded. The visiting time as recorded by the system may be not fully reliable because it might be that respondents opened the website, but did not use it while it remained open. Moreover, visiting time also depends on the bit rate of the individual respondent’s Internet.

In our intervention, exposure to the program was similar for both the sequential and the simultaneous group, although respondents in the simultaneous condition had the possibility of receiving more parts of the program than respondents in the sequential condition did. It may be that respondents in the simultaneous condition scanned the advice and did not read it carefully, resulting in comparable visiting times for the 2 intervention modes. In our sequential intervention, the modules were delivered sequentially over time so respondents could only choose 1 lifestyle module in the first year [27]. Exposure to multiple behaviors directly at the beginning of an intervention could have advantages for those people who drop out early [25]. Those participating in this way will already have received more information during their first visit than those taking part in a single-behavior intervention in which the people who drop out after their first visit can only receive information about a single behavior. Thus, it may be better to give respondents the choice of immediately initiating more than 1 module. A study by Brouwer et al [72] showed that more than half of their respondents selected 2 of 3 modules and approximately one-third initiated all 3 modules. A preference-based tailoring strategy [73,74]—a combination of our sequential and simultaneous condition—could be used in which respondents can select the modules they want to complete without any limitations on the number of behavior modules. The finding that revisits are uncommon in interventions [37,75]—our respondents visited the intervention on average only twice—is a further argument against delivering the modules, or even sections of particular modules, sequentially over time.

### Strengths and Limitations

To our knowledge, this study is unique in comparing the effectiveness of sequential and simultaneous interventions in addressing the 5 lifestyle behaviors of physical activity, fruit consumption, vegetable consumption, alcohol intake, and smoking. The intervention was effective with a small ES and was appreciated well. Our intervention met a number of criteria related to higher effectiveness of health behavior change interventions offered via the Internet, such as extensive use of theory and behavior change techniques [20]. However, several limitations should be kept in mind: dropout rates were high resulting in a small sample size; the findings were based on self-reports which may have resulted in recall bias (eg, the high proportion of respondents who reported being sufficiently physically active at baseline [87%] may represent an overestimation of their actual level of physical activity; however, another reason for this high proportion might be that the intensity of the different kinds of physical activities was not measured in the short version of the SQUASH that we used [41]); we cannot guarantee that a representative sample of the Dutch population was reached by our recruitment strategies; and a selective group filled out the follow-up questionnaires. Thus, the results may not be generalizable and may be biased. An additional limitation of our study is that respondents in the sequential condition had the possibility of choosing a maximum of 2 behavioral modules, whereas respondents in the simultaneous condition could receive personal feedback on more than 2 behaviors. Thus, the possible number of behavior modules that could be completed differed between the sequential and the simultaneous group, which implies that the sequential condition might have been more effective if respondents had the opportunity to choose more than 2 modules. Finally, the control condition received a minimal intervention, which might have led to improved lifestyle behaviors in this condition too.

### Implications for Future Interventions and Research

The high prevalence of people engaging in multiple health risk behaviors calls for the development of multiple behavior change interventions. Future studies should examine the number of behaviors that can be addressed in a multiple behavior change intervention without overloading the respondents [76]. Research is needed to identify strategies to stimulate exposure and participation, in particular for Internet interventions with multiple sessions, such as those involving email/phone contact with visitors and updates of the intervention website [39,42,77]. In addition to the necessity of research regarding the optimal delivery mode of these kinds of interventions, future research should focus on attracting, engaging, and retaining participants. In future interventions, other features could be added, such as short message service (SMS) text messaging, which can increase intervention effectiveness [20], or real-time sensors, such as accelerometer apps so that real-time motivational feedback can be provided [78]. Online community features, as a kind of self-help option, could be integrated in Web-based lifestyle programs to reduce attrition [79]. Moreover, interventions should be accessible on all different kinds of channels, such as desktop computers, laptops, smartphones, and tablets. Research is needed to assess the additional effects of these elements.

### Conclusions

Both sequential and simultaneous strategies were effective in improving lifestyle in a Web-based computer-tailored intervention. Because no crucial differences have been found with regard to dropout rates and appreciation of the interventions, providers can use the strategy that suits their particular circumstances best. However, the best kind of intervention may be dependent on the behavior that is targeted or other personal factors (eg, motivational level to change).

## Multimedia Appendix 1

T0-T1 completers and T0-T2 completers.

## Multimedia Appendix 2

Fully complete cases.

## Multimedia Appendix 3

Dropout analyses.

## Multimedia Appendix 4

CONSORT-EHEALTH checklist V1.6.2 [80].

## Abbreviations

BMI: body mass index
ES: effect size
FFQ: food frequency questionnaire
HRA: health risk appraisal
K10: Kessler psychological distress scale (10 items)
QFV: quantity-frequency-variability
SQUASH: short questionnaire to assess health-enhancing physical activity
