# Supplementary material for: Effects of a Web-Based Tailored Multiple-Lifestyle Intervention for Adults: A Two-Year Randomized Controlled Trial Comparing Sequential and Simultaneous Delivery Modes
Source: J Med Internet Res. 2014 Jan 27;16(1):e26. doi: 10.2196/jmir.3094 (PMC3936298; doi:10.2196/jmir.3094)
Supplement: Supplementary file 1 [file jmir_v16i1e26_app1.pdf]

Multimedia Appendix 2

T0-T1 completers and T0-T2 completers

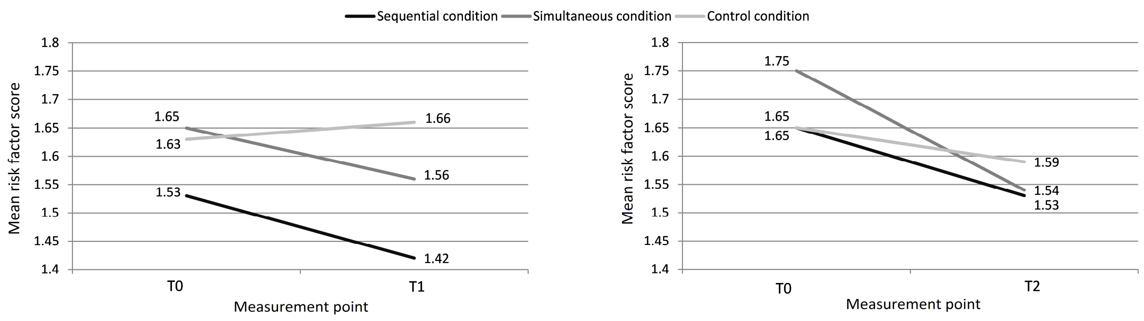

Figure 3: *Mean number of risk factors among the different conditions (T1: n = 791; T2: n = 1,128) at baseline and follow-up at T1 and T2 respectively.*

Table 5: *Results of the linear regression analysis (top-down procedure method [1]) with the risk factor index after 12 months (n = 768) and after 24 months (n = 1,113) as dependent variable*

	After 12 months (T1)				After 24 months (T2)			
**Variable**	**β**	***P***	**CI**	**ES**	**β**	***P***	**CI**	**ES**
Condition		**<.001*** **				**<.001*** **		
SeqC vs. CC [2]	-.10	**.003** **	-.35 - -.07	0.26	-.04	.19	-.20 - .04	0.10
SimC vs. CC [2]	-.07	**.03***	-.30 - -.02	0.20	-.06	**.03***	-.25 - -.01	0.16
SeqC vs. SimC [3]	-.02	.48	-.20 - .09	0.07	.02	.44	-.08 - .17	0.06
Age	-.06	.047**	-.01 - -.00	0.01	-.11	<.001***	-.01 - -.005	0.01
Education (low vs. high)	-.08	.02*	-.47 - -.04	0.32	---	---	---	---
Education (med vs. high)	-.03	.40	-.18 - .07	0.07	---	---	---	---
Income (low vs. high)	.07	.06†	-.01 - .34	0.21	.07	.02*	.03 - .32	0.21
Income (med vs. high)	.03	.44	-.09 - .20	0.07	.04	.21	-.04 - .19	0.09
Work situation	---	---	---	---	-.05	.04*	-.24 - -.009	0.15
K10	-.06	.07†	-.02 - .001	0.01	---	---	---	---
Physical activity (min)	---	---	---	---	-.09	<.001***	-.001 - .000	0.001
Vegetable intake (g)	-.21	<.001***	-.003 - -.002	0.002	-.22	<.001***	-.003 - -.002	0.004
Fruit intake (pieces)	-.30	<.001***	-.29 - -.19	0.30	-.29	<.001***	-.28 - -.20	0.29
Alcohol intake (glasses)	.25	<.001***	.11 - .18	0.18	.27	<.001***	.16 - .23	0.24
Smoking (cigarettes)	.25	<.001***	.04 - .07	0.07	.20	<.001***	.03 - .05	0.05
R^2				*.37*				*.36*

*†P < .10; *P < .05; **P < .01; ***P < .001*

[1] All variables regarding demographics, health status and lifestyle behaviour were included in the most extensive model

[2] The control condition was the reference category

[3] The simultaneous condition was the reference category

Table 6: *Results of the logistic regression analysis (top-down procedure [1]) with compliance with the five different guidelines (yes = 1; no = 0) after 12 months (n ≥ 760) and after 24 months (n ≥ 1,106) as dependent variables*

Lifestyle behaviour	***Group***	***T0-T1-completers***				***T0-T2-completers***			
		OR for change	***P***	***95% CI***	***ES***	***OR for change***	***P***	***95% CI***	***ES***
Phys. act.	Condition		.36				.71		
	SeqC vs. CC [2]	1.53	.19	.81 – 2.89	0.23	.96	.86	.57 – 1.61	0.02
	SimC vs. CC [2]	1.36	.30	.76 – 2.45	0.17	1.18	.52	.71 – 1.95	0.09
	SeqC vs.SimC [3]	1.12	.74	.57 – 2.21	0.06	.81	.43	.48 – 1.37	0.12
Vegetable	Condition		**.09†**				.14		
	SeqC vs. CC [2]	1.58	**.03***	1.05 – 2.39	0.25	1.15	.42	.82 – 1.63	0.08
	SimC vs. CC [2]	1.34	.18	.88 – 2.03	0.16	1.40	**.048***	1.00 – 1.94	0.18
	SeqC vs.SimC [3]	1.18	.44	.78 – 1.81	0.09	.83	.28	.58 – 1.17	0.11
Fruit	Condition		.15				**.04***		
	SeqC vs. CC [2]	1.48	**.06†**	.99 – 2.23	0.22	.94	.71	.67 – 1.32	0.04
	SimC vs. CC [2]	1.28	.22	.86 – 1.91	0.14	1.43	**.04***	1.02 – 2.01	0.20
	SeqC vs.SimC [3]	1.16	.49	.76 – 1.76	0.08	.66	**.02***	.46 - .94	0.23
Alcohol	Condition		.17				**.08†**		
	SeqC vs. CC [2]	1.38	.28	.77 – 2.47	0.18	1.75	**.03***	1.07 – 2.86	0.31
	SimC vs. CC [2]	1.72	**.07†**	.97 – 3.05	0.30	1.31	.25	.83 – 2.08	0.15
	SeqC vs.SimC [3]	.80	.50	.43 – 1.51	0.12	1.34	.27	.80 – 2.24	0.16
Smoking	Condition		.34				**.02***		
	SeqC vs. CC [2]	.83	.67	.34 – 2.00	0.10	1.52	.22	.78 – 2.94	0.23
	SimC vs. CC [2]	.55	.15	.24 – 1.25	0.33	.60	**.07†**	.35 – 1.04	0.28
	SeqC vs.SimC [3]	1.50	.33	.66 – 3.44	0.22	2.52	**.005****	1.32 – 4.82	0.51

*†P < .10; *P < .05; **P < .01*

[1] All variables regarding demographics, health status and lifestyle behaviour were included in the most extensive model

[2] The control condition was the reference category

[3] The simultaneous condition was the reference category
