# Supplementary material for: Effects of a Web-Based Tailored Multiple-Lifestyle Intervention for Adults: A Two-Year Randomized Controlled Trial Comparing Sequential and Simultaneous Delivery Modes
Source: J Med Internet Res. 2014 Jan 27;16(1):e26. doi: 10.2196/jmir.3094 (PMC3936298; doi:10.2196/jmir.3094)
Supplement: Supplementary file 2 [file jmir_v16i1e26_app2.pdf]

Multimedia Appendix 3

Fully complete cases

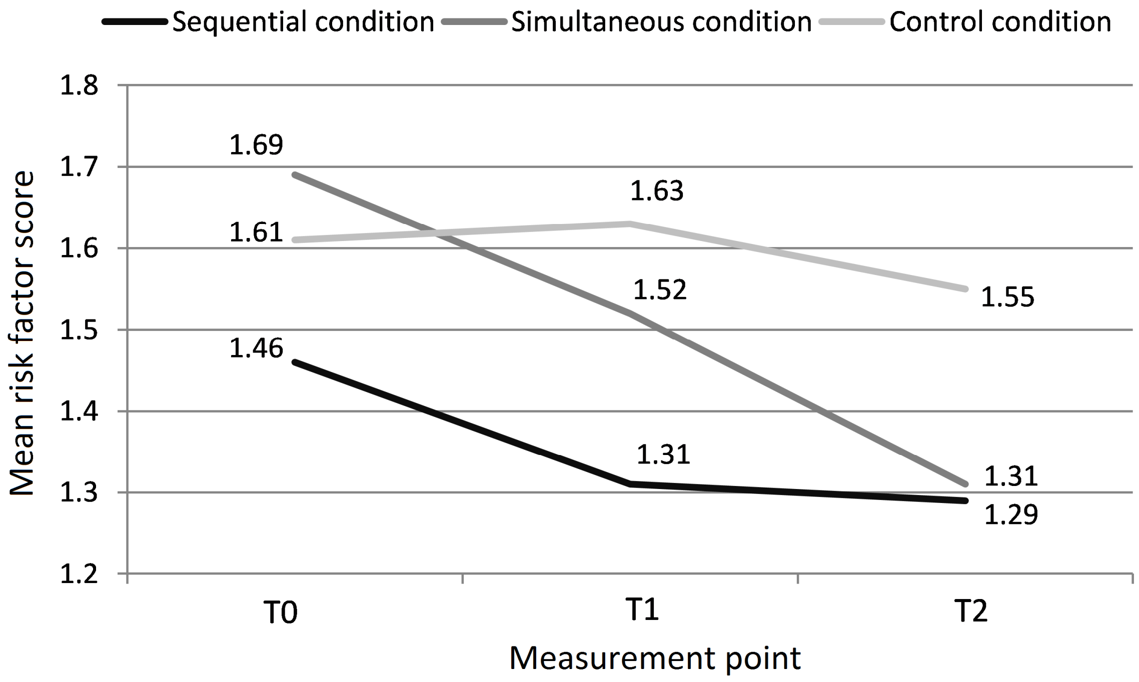

Figure 4: *Mean number of risk factors among the different conditions (complete cases: n = 475) at baseline and follow-up (sequential condition n = 140; simultaneous condition n = 143; control condition n = 192)*

Table 7: *Results of the linear regression analysis (top-down procedure) with the risk factor index after 12 months and after 24 months as dependent variable (n = 466)*

	After 12 months (T1)				After 24 months (T2)			
**Variable**	**β**	***P***	**CI**	**ES**	**β**	***P***	**CI**	**ES**
Condition		**<.001**				**<.001**		
SeqC vs. CC	-.11	**.006****	-.42 - -.07	0.31	-.09	**.03***	-.38 - -.03	0.26
SimC vs. CC	-.09	**.03***	-.36 - -.02	0.24	-.14	**.001****	-.48 - -.13	0.39
SeqC vs. SimC	-.02	.59	-.24 - .13	0.07	.05	.27	-.08 - .29	0.13
Age	-.07	.049*	-.01 - .000	0.01	---	---	---	---
Education (low vs. high)	-.08	.04*	-.58 - -.02	0.38	---	---	---	---
Education (med vs. high)	-.04	.36	-.23 - .08	0.09	---	---	---	---
Income (low vs. high)	.09	.048*	.002 - .42	0.27	.09	.03*	.02 - .43	0.28
Income (med vs. high)	.07	.11	-.03 - .32	0.18	.11	.02*	.04 - .38	0.27
Work situation	---	---	---	---	-.07	.08†	-.30 - .02	0.18
Physical activity (min)	-.06	.09†	-.001 - .000	0.00	-.09	.02*	-.001 - .000	0.001
Vegetable intake (g)	-.25	<.001***	-.004 - -.002	0.003	-.27	<.001***	-.004 - -.002	0.004
Fruit intake (pieces)	-.30	<.001***	-.31 - -.19	0.31	-.31	<.001***	-.31 - -.19	0.32
Alcohol intake (glasses)	.31	<.001***	.17 - .28	0.28	.26	<.001***	.14 - .24	0.24
Smoking (cigarettes)	.27	<.001***	.05 - .08	0.08	.22	<.001***	.03 - .07	0.06
R^2				*.43*				*.40*

*†P < .10; *P < .05; **P < .01; ***P < .001*

[1] All variables regarding demographics, health status and lifestyle behaviour were included in the most extensive model

[2] The control condition was the reference category

[3] The simultaneous condition was the reference category

Table 8: *Results of the logistic regression analysis (top-down procedure) with compliance with the five different guidelines (yes=1; no=0) after 12 months and after 24 months as dependent variables (n=457)*

Lifestyle behaviour	***Group***	**After 12 months (T1)**				**After 24 months (T2)**			
		OR for change	***P***	***95% CI***	***ES***	***OR for change***	***P***	***95% CI***	***ES***
Phys. act.	Condition		.82				.31		
	SeqC vs. CC [2]	1.32	.53	.55 – 3.15	0.15	.76	.58	.28 – 2.03	0.15
	SimC vs. CC [2]	1.05	.90	.48 – 2.30	0.03	1.76	.26	.66 – 4.68	0.31
	SeqC vs.SimC [3]	1.26	.50	.46 – 3.15	0.13	.43	.14	.14 – 1.31	0.46
Vegetable	Condition		**.06**†				**.06**†		
	SeqC vs. CC [2]	1.69	**.05**†	1.00 – 2.86	0.29	1.06	.84	.62 – 1.81	0.03
	SimC vs. CC [2]	1.75	**.04***	1.03 – 2.98	0.31	1.83	**.03***	1.08 – 3.11	0.33
	SeqC vs.SimC [3]	.96	.89	.56 – 1.66	0.02	.58	**.06**†	.33 – 1.02	0.30
Fruit	Condition		.55				**.07**†		
	SeqC vs. CC [2]	1.23	.44	.73 – 2.09	0.12	1.67	**.06**†	.97 – 2.85	0.28
	SimC vs. CC [2]	1.32	.30	.78 – 2.21	0.15	1.70	**.048***	1.00 – 2.88	0.29
	SeqC vs.SimC [3]	.94	.82	.54 – 1.64	0.04	.98	.94	.55 – 1.74	0.01
Alcohol	Condition		**.07**†				**.003**		
	SeqC vs. CC [2]	2.77	**.02***	1.16 – 6.65	0.56	3.85	**.002****	1.62 – 9.16	0.74
	SimC vs. CC [2]	1.66	.19	.77 – 3.58	0.28	2.68	**.01***	1.23 – 5.84	0.54
	SeqC vs.SimC [3]	1.67	.28	.65 – 4.26	0.28	1.44	.45	.57 – 3.65	0.20
Smoking	Condition		.27				.27		
	SeqC vs. CC [2]	.67	.53	.19 – 2.36	0.22	1.37	.62	.40 – 4.65	0.17
	SimC vs. CC [2]	.41	.11	.13 – 1.24	0.50	.56	.25	.21 – 1.51	0.32
	SeqC vs.SimC [3]	1.64	.39	.53 – 5.08	0.27	2.44	.13	.76 – 7.83	0.49

†*P* < .10; **P* < .05; ***P* < .01

[1] *All variables regarding demographics, health status and lifestyle behaviour were included in the most extensive model*

[2] *The control condition was the reference category*

[3] *The simultaneous condition was the reference category*
