# Supplementary material for: Effects of a Web-Based Tailored Multiple-Lifestyle Intervention for Adults: A Two-Year Randomized Controlled Trial Comparing Sequential and Simultaneous Delivery Modes
Source: J Med Internet Res. 2014 Jan 27;16(1):e26. doi: 10.2196/jmir.3094 (PMC3936298; doi:10.2196/jmir.3094)
Supplement: Supplementary file 3 [file jmir_v16i1e26_app3.pdf]

Multimedia Appendix 1

Drop-out analyses

Table 4: *Results of the drop-out analyses*

Variable [a]	***Differences T1C vs. NC*** [b]	***Differences T2C vs. NC*** [b]	***Differences FCC vs. NC*** [b]
**Demographics**			
**Age** (19-65)	T1C > NC	T2C > NC	FCC > NC
**Employment situation** n = 4,970	T1C < NC	T2C < NC	FCC < NC
Job (paid employment)			
No job			
**Marital status** n = 4,973	T1C > NC	T2C > NC	FCC > NC†
Relationship			
Single			
# persons in household n = 4,980	T1C < NC	T2C < NC	FCC < NC
**Native country** n = 4,973	T1C > NC		
The Netherlands			
Other			
**Health status**			
**BMI (13.82-58.11)** n = 5,012		T2C > NC	
**Psychol. distress (12-50)** n = 4,944			FCC > NC†
**Disease(s)** n = 4,950			
Diabetes	T1C > NC	T2C > NC†	
Brain haemorrhage, TIA			
Heart attack	T1C > NC	T2C > NC	FCC > NC
Other serious heart disease	T1C > NC†	T2C > NC	FCC > NC
Cancer	T1C > NC†		
High blood pressure	T1C > NC	T2C > NC	FCC > NC
Asthma, COPD			
One or more diseases	T1C > NC	T2C > NC	FCC > NC
**Lifestyle behavior**			
Mean number of risk factors	T1C < NC	T2C < NC	FCC < NC
**Physical activity** n = 5,053			
Mean number of minutes per day	T1C > NC		FCC > NC†
Non-compliance	T1C < NC		FCC < NC
**Vegetable consumption** n = 5,018			
Mean number of grams	T1C > NC	T2C > NC	FCC > NC
Non-compliance	T1C < NC	T2C < NC	FCC < NC
**Fruit consumption** n = 5,019			
Mean pieces of fruit	T1C > NC	T2C > NC	FCC > NC
Non-compliance	T1C < NC	T2C < NC	FCC < NC
**Alcohol intake** n = 5,034			
Mean number of drinks		T2C < NC	FCC < NC
Non-compliance	T1C < NC†	T2C < NC	FCC < NC†
**Smoking** n = 5,055			
Mean number of cigarettes	T1C < NC	T2C < NC	FCC < NC
Non-compliance	T1C < NC	T2C < NC	FCC < NC

Note: † *P* < .10

[a] No statistically significant differences were found with regard to gender, educational level, income, and quality of life.

[b] T1C = respondents who completed the T1-measurement; T2C = respondents who completed the T2-measurement; FCC = respondents who completed the T1- and the T2-measurement (fully complete cases); NC = respondents who did not complete the T1 or T2-measurement; a comma (“,”) means that the scores of the different conditions are equal; the “>” sign means that respondents in the first group (in front of the sign) were significantly older, more often in relationship, Dutch, having a higher BMI, suffering from a disease and having a healthier lifestyle ; the “<” sign means that respondents in the first group (in front of the sign) were significantly less often employed, lived with fewer peoples in one household, reported a lower risk factor score and less non-compliance with guidelines.
