# Supplementary material for: Effects of a Web-Based Tailored Multiple-Lifestyle Intervention for Adults: A Two-Year Randomized Controlled Trial Comparing Sequential and Simultaneous Delivery Modes
Source: J Med Internet Res. 2014 Jan 27;16(1):e26. doi: 10.2196/jmir.3094 (PMC3936298; doi:10.2196/jmir.3094)
Supplement: Supplementary file 4 [file jmir_v16i1e26_app4.pdf]

CONSORT-EHEALTH Checklist V1.6.2 Report

(based on CONSORT-EHEALTH V1.6), available at [http://tinyurl.com/consort-ehealth-v1-6].

3094

Date completed
11/9/2013 11:14:24
by
Daniela Schulz

Effects of a web-based tailored multiple lifestyle intervention for adults: A two-year randomized controlled trial comparing sequential and simultaneous delivery modes

TITLE

1a-i) Identify the mode of delivery in the title
YES:
"[...] web-based tailored multiple lifestyle intervention [...]"
1a-ii) Non-web-based components or important co-interventions in title
n/a
1a-iii) Primary condition or target group in the title
YES:
"[...] lifestyle intervention for adults"

ABSTRACT

1b-i) Key features/functionalities/components of the intervention and comparator in the METHODS section of the ABSTRACT
YES:
"In a health risk appraisal, all respondents (N=5,055) received feedback on their lifestyle behaviors that indicate whether they complied with the Dutch guidelines for physical activity, vegetable consumption, fruit consumption, alcohol intake, and smoking. Participants in the sequential and simultaneous condition additionally received tailored motivational feedback to change unhealthy behaviors one at a time (sequential) or all at the same time (simultaneous)."
1b-ii) Level of human involvement in the METHODS section of the ABSTRACT

1b-iii) Open vs. closed, web-based (self-assessment) vs. face-to-face assessments in the METHODS section of the ABSTRACT

1b-iv) RESULTS section in abstract must contain use data
YES:
"In a health risk appraisal, all respondents (N=5,055) received feedback on their lifestyle behaviors that indicate whether they complied with the Dutch guidelines for physical activity, vegetable consumption, fruit consumption, alcohol intake, and smoking. Participants in the sequential (n = 1,736) and simultaneous (n = 1,638) condition additionally received tailored motivational feedback to change unhealthy behaviors one at a time (sequential) or all at the same time (simultaneous)."

"The sequential condition resulted in the most significant effects in comparison to the control condition after 12 months (effect size = 0.28). After 24 months, the simultaneous condition was most effective (effect size = 0.18). All five individual lifestyle behaviors changed over time, but only a few effects differed significantly between the conditions: at both follow-up measurements the sequential condition was found to result in significant changes in smoking abstinence in comparison with the simultaneous condition (effect size T1 = 0.31; effect size T2 = 0.41); after 24 months, the sequential condition was more effective in decreasing alcohol consumption than the control condition (effect size = 0.27). Change was further predicted by the amount of exposure to the intervention (total visiting time: $\beta = -.06$; $P = .01$; total number of visits: $\beta = -.11$; $P < .001$)."
1b-v) CONCLUSIONS/DISCUSSION in abstract for negative trials
"Providers can use the strategy that suits their particular circumstances best. However, the best kind of intervention may depend on the behavior that is targeted or personal preferences."

INTRODUCTION

2a-i) Problem and the type of system/solution
Our intervention is intented as a stand-alone intervention for adults of the general population; however, it was connected to the Adult Health Monitor of different Regional Health Authorities in the Netherlands.

"Given the high prevalence of unhealthy lifestyle habits [5-8], it is reasonable to offer interventions that can be disseminated among large numbers of people at low cost [9]. As individuals with multiple unhealthy lifestyle behaviors are at the greatest risk of developing chronic diseases, leading to increased health care costs [10], web-based interventions with a focus on different lifestyle behaviors and integrated within one intervention seem to be an appropriate choice. To increase changes in health behavior, tailored interventions using a computerized expert system to select the best fitting messages in order to generate personal relevant feedback messages have been developed [11]. Previous studies have shown that web-based computer-tailored interventions are an effective tool for improving health-related behaviors [e.g., 12-16]. Moreover, web-based computer-tailored interventions which contain relevant and attractive information adapted to the respondents' individual characteristics and needs have proven to be cost-effective [e.g. 17,18] and have been evaluated more positively than general information [8].
It has been suggested that interventions that focus on multiple behaviors have a greater impact on public health than single-behavior interventions [8,19]. However, such interventions are more extensive and thus require more engagement, time, and effort from the respondent. There is limited and inconsistent evidence about how best to accomplish multiple behavior change when using web-based computer-tailored interventions."
2a-ii) Scientific background, rationale: What is known about the (type of) system

"There is limited and inconsistent evidence about how best to accomplish multiple behavior change when using web-based computer-tailored interventions. One strategy is to intervene in one behavior at a time (sequential approach); another approach is to intervene in all health risk behaviors at the same time (simultaneous approach) [8,20-26]. In earlier studies, no consistent findings were reported regarding the most effective strategy. For example, King et al. [21] and Hyman et al. [20] reported that their simultaneous intervention mode was superior to their sequential intervention mode. The first aimed at changes in diet and physical activity; the latter aimed at smoking cessation and improvements in diet and physical activity. A study by Vandelanotte et al. [26] aimed at lowering fat intake and increasing physical activity found no differences between the sequential and the simultaneous condition.
Focusing on different behaviors sequentially at different points in time is associated with less cognitive effort during the individual visits; however, respondents may experience lower levels of autonomy due to the limited choices during the individual visits [27,28], and repeated participation is necessary to receive information about multiple behaviors. Intervening in all behaviors simultaneously has the advantage that respondents receive all relevant information during the first visit; however, such a program may lead to ego-depletion by overwhelming respondents with too much information [29,30] which furthermore may lead to a more negative feeling regarding the intervention and immediate drop-out. In previous research, dose-response relationships have been found between exposure to an intervention (number and duration of exposures) and behavior change outcomes [31,32]. This may imply that repeated participation and thus repeated exposure to the web-based program can be beneficial for realizing substantial behavioral change [33]. Appreciation of the intervention may lead to increased use, which in turn may improve intervention effectiveness [34-36]. Thus, both delivery modes have potential advantages and disadvantages which may influence their effectiveness, use, and the appreciation of the different types of interventions. Current evidence regarding the effectiveness of sequential and simultaneous delivery modes is inconsistent and to date none of these studies have tested the two delivery modes within an intervention targeting five different health behaviors."

METHODS

3a) CONSORT: Description of trial design (such as parallel, factorial) including allocation ratio

"[...] the main aim of the current study was to assess whether a multisession, web-based tailored lifestyle intervention was effective in enhancing multiple lifestyle behaviors (i.e., physical activity, vegetable consumption, fruit consumption, alcohol intake, smoking) in the long term. First, potential differences in effects of lifestyle change in general were assessed between the sequential and simultaneous delivery mode and a control condition at 12-month and 24-month follow-up. Second, we evaluated whether there were differences between the three groups with regard to effects on adherence to individual guidelines for each of the five behaviors being assessed. As a tertiary objective, a process evaluation was executed by studying the influence of intervention exposure on effectiveness and by summarizing the appreciation of the intervention."

3b) CONSORT: Important changes to methods after trial commencement (such as eligibility criteria), with reasons

n/a = no changes

3b-i) Bug fixes, Downtimes, Content Changes

We sent additional (not planned) reminders in order to increase the response.

4a) CONSORT: Eligibility criteria for participants

"The following inclusion criteria were established for this study: being between 18 and 65 years old, having a computer with Internet access, having basic Internet literacy, and having a valid e-mail address."

4a-i) Computer / Internet literacy

"The following inclusion criteria were established for this study: [...] having basic Internet literacy [...]"

4a-ii) Open vs. closed, web-based vs. face-to-face assessments:

"In first instance, adult participants were recruited via four Dutch Regional Health Authorities that had conducted the quadrennial Adult Health Monitor 2009 of inhabitants of the provinces of North-Brabant and Zeeland. [...] At the end of this web-based questionnaire, participants were invited to take part in our intervention study. [...] The website was also open to the general public, which means that it was also possible to register directly on the website without having to complete the Monitor."

4a-iii) Information giving during recruitment

"[...] At the end of this web-based questionnaire, participants were invited to take part in our intervention study. They had to give informed consent and provide their e-mail address for participation and the handling of their data. Three weeks later, the study sample received an e-mail containing an invitation and a link to the intervention website."

4b) CONSORT: Settings and locations where the data were collected

"The web-based intervention, focusing on unhealthy lifestyle behaviors in the general population, was conducted in the Netherlands from November 2009 until July 2012. Follow-up measurements took place 12 and 24 months after the first intervention visit. In first instance, adult participants were recruited via four Dutch Regional Health Authorities that had conducted the quadrennial Adult Health Monitor 2009 of inhabitants of the provinces of North-Brabant and Zeeland. [...] The website was also open to the general public, which means that it was also possible to register directly on the website without having to complete the Monitor."

4b-i) Report if outcomes were (self-)assessed through online questionnaires

"The intervention program, called "myHealthyBehavior" (Dutch: mijnGezondGedrag), was a web-based computer-tailored program targeting adults. [...] In this part, self-assessed questionnaires were used to measure the psychosocial concepts of the I-Change model."

4b-ii) Report how institutional affiliations are displayed

5) CONSORT: Describe the interventions for each group with sufficient details to allow replication, including how and when they were actually administered

5-i) Mention names, credential, affiliations of the developers, sponsors, and owners

The different study groups (sequential, simultaneous, control) are described in the method section.

5-ii) Describe the history/development process

We had executed a pilot study in another province in the Netherlands (in Limburg).

5-iii) Revisions and updating

n/a - no major changes

5-iv) Quality assurance methods

5-v) Ensure replicability by publishing the source code, and/or providing screenshots/screen-capture video, and/or providing flowcharts of the algorithms used

5-vi) Digital preservation

5-vii) Access

Respondents of the Adult Health Monitor 2009 were allowed to take part in our study. However, the website was also open to the general public. Participation was free of charge and respondents are eligible to win prizes (see study protocol: Schulz DN, Kremers SP, Van Osch LA, Schneider F, Van Adrichem MJ, De Vries H. Testing a Dutch web-based tailored lifestyle program among adults: a study protocol. BMC Public Health 2011;11:108. PMID: 21324181).

5-viii) Mode of delivery, features/functionalities/components of the intervention and comparator, and the theoretical framework
"The intervention program, called “myHealthyBehavior” (Dutch: mijnGezondGedrag), was a web-based computer-tailored program targeting adults. The main aim of the intervention was to motivate participants to enhance five health behaviors. The theoretical framework for the development of the intervention was the I-Change model [39]. In the first part of the intervention, all groups received a health risk appraisal (HRA) in which the five behaviors were placed in the context of the following Dutch public health guidelines for each of the different behaviors: [...] The second part of the intervention was only available for the two of the experimental conditions: (1) the sequential condition (SeqC) and (2) the simultaneous condition (SimC). In this part, self-assessed questionnaires were used to measure the psychosocial concepts of the I-Change model. The feedback messages were, in turn, based on these questionnaires and were revealed on the respondents’ computer screens immediately after the completion of the surveys. Respondents who did not comply with at least one guideline were invited to change the behavior which they had not complied with. They were invited to complete additional questions regarding motivational constructs related to a chosen behavior (sequential condition) or all relevant behaviors (simultaneous condition). Motivational feedback was given that was related to the relevant behaviors. Feedback on each person’s perception of the pros and cons of the health behavior (attitudinal feedback) was given as the first step of the program, followed by feedback on perceived social influences as the second step. For example, information was given on how the social environment could help the respondent to live healthily. In the third step, the concept of preparatory planning was addressed. Feedback was provided on how to prepare for behavior change, for example by planning to be physically active at fixed times, adding the plans to an agenda, trying new/different kinds of sports, having enough vegetables in stock at home, eating fruit or vegetables as a snack, and (for smokers) planning a quit date. In the final step, the focus was placed on how to cope with difficult situations in order to also increase self-efficacy. Several tailoring strategies were used in the feedback messages; for example, respondents were addressed by their name, normative and ipsative feedback were given (i.e., during re-visits current scores were compared to scores of previous visits), graphs and bar charts were included, and a personal tone and empathy was applied."
5-ix) Describe use parameters
On the website, respondents could find information about the number of e-mails/invitations they will receive and about the duration of the study.
5-x) Clarify the level of human involvement

5-xi) Report any prompts/reminders used
"At the end of this web-based questionnaire, participants were invited to take part in our intervention study. They had to give informed consent and provide their e-mail address for participation and the handling of their data. Three weeks later, the study sample received an e-mail containing an invitation and a link to the intervention website. After one month, a reminder e-mail was sent to the individuals in the sample who had not responded to the first invitation."
5-xii) Describe any co-interventions (incl. training/support)
n/a - Our intervention was a stand-alone intervention, with a connection to the Adult Health Monitor of different Regional Health Authorities.
6a) CONSORT: Completely defined pre-specified primary and secondary outcome measures, including how and when they were assessed
The following (outcome) measures were included:

- Demographic information & health status
- Lifestyle behaviors: physical activity, fruit consumption,
vegetable consumption, alcohol intake, smoking
- Lifestyle risk factor
- Number of logins to the tailored intervention
- Time respondents spent on the tailored intervention
- Appreciation of the intervention program

6a-i) Online questionnaires: describe if they were validated for online use and apply CHERRIES items to describe how the questionnaires were designed/deployed

6a-ii) Describe whether and how “use” (including intensity of use/dosage) was defined/measured/monitored
"To report user statistics, we recorded the number of logins to the tailored intervention per respondent as well as the time respondents spent on the tailored intervention during their unique visits."
6a-iii) Describe whether, how, and when qualitative feedback from participants was obtained
We added a feedback questionnaire on the website which could be filled in at every moment. Moreover, we received some e-mails from respondents.
6b) CONSORT: Any changes to trial outcomes after the trial commenced, with reasons
n/a - no changes
7a) CONSORT: How sample size was determined
7a-i) Describe whether and how expected attrition was taken into account when calculating the sample size
"As the linear mixed model analysis (lifestyle factor is the outcome variable) was the main analysis, power analysis suggested that a total sample of 1,182 respondents was needed (correcting for possible attrition) based on p = .05, a power of 80%, a two-sided test, and an effect size (ES) of 0.20."
7b) CONSORT: When applicable, explanation of any interim analyses and stopping guidelines
n/a
8a) CONSORT: Method used to generate the random allocation sequence
"Randomization to one of the three study groups took place by means of a computer software randomization system."
8b) CONSORT: Type of randomisation; details of any restriction (such as blocking and block size)
"No block or cluster randomization was applied; the randomization was done at the individual level."
9) CONSORT: Mechanism used to implement the random allocation sequence (such as sequentially numbered containers), describing any steps taken to conceal the sequence until interventions were assigned
Also the control group got a small amount of personlized information in order to conceal allocation to the control group.
10) CONSORT: Who generated the random allocation sequence, who enrolled participants, and who assigned participants to interventions
Participants of the Adult Health Monitor were allowed to take part in our study. The computer-tailoring software, facilitated by the software-company, was used for the randomization process.
11a) CONSORT: Blinding - If done, who was blinded after assignment to interventions (for example, participants, care providers, those assessing outcomes) and how
11a-i) Specify who was blinded, and who wasn’t
Our respondents were blinded.
11a-ii) Discuss e.g., whether participants knew which intervention was the “intervention of interest” and which one was the “comparator”

11b) CONSORT: If relevant, description of the similarity of interventions
n/a

12a) CONSORT: Statistical methods used to compare groups for primary and secondary outcomes
"The data were analysed using SPSS software, version 20. To examine whether the randomization had been successful, the three groups were compared in terms of demographics, health status, and lifestyle behavior; ANOVAs were executed for continuous variables and chi-square tests for discrete variables. In the case of significant differences, variables were included as covariates (i.e., potential confounders) in subsequent analyses. Descriptive statistics were used to describe the characteristics of the study sample and the drop-out rate within the groups. Dropout analyses, including a comparison between respondents lost to follow-up and T0-T1 completers and T0-T2 completers, respectively, and fully complete cases have also been done by using ANOVAs and chi-square tests.
We calculated a risk factor score by summing up all risky/unhealthy behaviors defined by the guideline status; the value for the risk factor score could range from 0 (adhering to all guidelines) to 5 (adhering to no guideline). The three study groups were compared in terms of their lifestyle behavior at the follow-up measurements. First, ES were calculated based on means and odds ratios (Cohen's d). ES below 0.30 were considered small, while those between 0.30 and 0.80 were considered medium, and those larger than 0.80 were regarded as large [52]. Second, repeated measures analyses, using the top-down procedure, were conducted to study changes during the study period (time) and differences in changes between the study groups (time × condition). Linear mixed model analyses were used for the analyses with the risk factor score as outcome measure. Logistic mixed model analyses were used with the guidelines status for the five lifestyle behaviors as outcome measures. These kinds of analyses allow for inclusion of all cases (despite missing values of the outcome variable), and are valid in case the missing values satisfy the missingness at random assumption [53].
For the sensitivity analyses, differences in effect between the groups were explored by means of linear and logistic regression analyses, using the top-down procedure. The dependent variables were (1) the risk factor score; (2a) complying with the physical activity guideline (yes = 1; no = 0); (2b) complying with the vegetable guideline (yes = 1; no = 0); (2c) complying with the fruit guideline (yes = 1; no = 0); (2d) complying with the alcohol guideline (yes = 1; no = 0); (2e) complying with the smoking guideline (yes = 1; no = 0). All analyses were done for both the 12-month and 24-month follow-up measurement. To increase power and external validity, these regression analyses were first performed for T0-T1 completers and T0-T2 completers, respectively. Next, these analyses were also performed based on fully complete cases (i.e., respondents who completed both follow-up measurements). The results of the sensitivity analyses are outlined in the Appendix.
Among the experimental conditions, linear regression analyses were performed to study the predictive value of the total visiting time of the intervention and the total number of visits during the study period on the risk factor score after 24 months. Descriptive statistics were used to describe the evaluation/appreciation of the intervention at different time points.
Tests were performed at $\alpha = .05$ for the intervention factor and $\alpha = .10$ for covariates [54]."
12a-i) Imputation techniques to deal with attrition / missing values
"[...] repeated measures analyses, using the top-down procedure, were conducted to study changes during the study period (time) and differences in changes between the study groups (time × condition). Linear mixed model analyses were used for the analyses with the risk factor score as outcome measure. Logistic mixed model analyses were used with the guidelines status for the five lifestyle behaviors as outcome measures. These kinds of analyses allow for inclusion of all cases (despite missing values of the outcome variable), and are valid in case the missing values satisfy the missingness at random assumption [53]." Furthermore, we used complete case analyses (different groups) as sensitivity analyses.
12b) CONSORT: Methods for additional analyses, such as subgroup analyses and adjusted analyses
As mentioned above, analyses including complete cases were performed as sensitivity analyses. Different groups (i.e., fully complete cases; T0-T1-completers; T0-T2-completers) were included.

RESULTS

13a) CONSORT: For each group, the numbers of participants who were randomly assigned, received intended treatment, and were analysed for the primary outcome
We added an attrition diagram in which these results are shown.
13b) CONSORT: For each group, losses and exclusions after randomisation, together with reasons
We added an attrition diagram in which these results are shown. We do not know the reasons for drop-outs.
13b-i) Attrition diagram
YES: we added an attrition diagram.
14a) CONSORT: Dates defining the periods of recruitment and follow-up
"The web-based intervention [...] was conducted in the Netherlands from November 2009 until July 2012. Follow-up measurements took place 12 and 24 months after the first intervention visit. "
14a-i) Indicate if critical "secular events" fell into the study period

14b) CONSORT: Why the trial ended or was stopped (early)
n/a
15) CONSORT: A table showing baseline demographic and clinical characteristics for each group
YES: see Table 1
15-i) Report demographics associated with digital divide issues
YES: see Table 1
16a) CONSORT: For each group, number of participants (denominator) included in each analysis and whether the analysis was by original assigned groups
16-i) Report multiple "denominators" and provide definitions
See Figure 1 (flow-chart)
16-ii) Primary analysis should be intent-to-treat
YES: we used mixed model analyses as primary analyses; and complete case analyses as secondary analyses.
17a) CONSORT: For each primary and secondary outcome, results for each group, and the estimated effect size and its precision (such as 95% confidence interval)
See Tables 2, 3, 5-8
17a-i) Presentation of process outcomes such as metrics of use and intensity of use
"Exposure to the intervention
When comparing the total visiting time and the total number of visits in the intervention during the 24 months, statistically significant differences between the three groups were found. On average, respondents in the sequential condition visited the intervention for 31 ± 40 minutes, respondents of the simultaneous condition stayed on the website for 28 ± 36 minutes; and respondents in the control condition visited the website for 16 ± 21 minutes ($F = 23.78$; $P < .001$; SeqC, SimC > CC). The mean number of visits in the sequential condition was 2.04 ± 1.35; in the simultaneous condition this was 2.01 ± 1.45; and in the control condition this was 1.85 ± .93 ($F = 2.84$; $P = .06$; SeqC, SimC > CC). The regression analyses conducted among respondents in the sequential and the simultaneous conditions only showed that the risk factor after 24 months was predicted by the total visiting time ($\beta = -.06$; $P = .01$) and the total number of visits during the study period ($\beta = -.11$; $P < .001$). Longer visits and a greater number of visits predicted more favorable risk factor changes."
17b) CONSORT: For binary outcomes, presentation of both absolute and relative effect sizes is recommended
See Tables 2, 3, 5-8

18) CONSORT: Results of any other analyses performed, including subgroup analyses and adjusted analyses, distinguishing pre-specified from exploratory

See results section

18-i) Subgroup analysis of comparing only users

See appendices and the following results:

"The sensitivity analyses showed similar results (see Appendices 2 and 3), except for two differences: (1) among the T0-T1 completers, both simultaneous and sequential interventions were effective in reducing the risk factor score after 12 months compared to the control condition; (2) among fully complete cases, both simultaneous and sequential interventions revealed significant effects in reducing the risk factor score in comparison to the control condition after 24 months."

"However, when performing the sensitivity analyses, no consistent pattern could be found. Differences in statistical significance were found, especially regarding comparisons between groups with regard to fruit and vegetable intake, alcohol consumption, and smoking. More information can be found in Appendices 2 and 3."

19) CONSORT: All important harms or unintended effects in each group

No intended harms were reported.

19-i) Include privacy breaches, technical problems

It might be that technical problems occured (in all groups).

19-ii) Include qualitative feedback from participants or observations from staff/researchers

"Some suggestions were given for improving the intervention; for example, attention should be paid to personal circumstances by asking more specific questions about reasons for not eating sufficient vegetables or for being insufficiently physically active, and the advice could be made more personal by giving more concrete examples."

DISCUSSION

20) CONSORT: Trial limitations, addressing sources of potential bias, imprecision, multiplicity of analyses

20-i) Typical limitations in ehealth trials

"However, several limitations should be kept in mind: drop-out rates were high resulting in a too small sample size; the findings were based on self-reports which may have resulted in recall bias (e.g., the high proportion of respondents who reported being sufficiently physically active at baseline [87%] may represent an overestimation of their actual level of physical activity); it might be that the intensity of the different kinds of physical activities was not measured in the short version of the SQUASH that we used [38]); and a selective group filled out the follow-up questionnaires. Thus, the results may not be generalizable and may be biased. In addition, the possible number of behavior modules that could be completed differed between the sequential and the simultaneous group, which implies that the sequential condition might have been more effective if respondents had the opportunity to choose more than two modules. Finally, the control condition received a minimal intervention which might have led to improved lifestyle behaviors in this condition, too."

21) CONSORT: Generalisability (external validity, applicability) of the trial findings

21-i) Generalizability to other populations

"The results of the sensitivity analyses, using regression techniques, might not be generalizable to the general population, but only to those people who remain participating in such a study over a longer period of time."

"[...] and a selective group filled out the follow-up questionnaires. Thus, the results may not be generalizable and may be biased."

21-ii) Discuss if there were elements in the RCT that would be different in a routine application setting

22) CONSORT: Interpretation consistent with results, balancing benefits and harms, and considering other relevant evidence

22-i) Restate study questions and summarize the answers suggested by the data, starting with primary outcomes and process outcomes (use)

"The main aim of this study was to evaluate the effectiveness of two tailored web-based interventions compared to a control condition with regard to lifestyle improvement. Overall, the computer-tailored multiple health behavior intervention resulted in favorable lifestyle changes. Compared to the control condition, the sequential delivery mode was found to be most effective after 12 months, whereas the simultaneous delivery mode was most effective after 24 months. The sensitivity analyses yielded comparable results and suggested slightly stronger effects for both delivery modes, which may be due to the fact that those motivated to fill out more post-measurements were also those more motivated to change. The effect sizes were small which is common for tailored interventions, but they can still result in a large public health impact when widely implemented [12,55]. Moreover, our control condition received a minimal intervention (i.e., the tailored HRA). Personalized information regarding one's lifestyle behavior may be sufficient to facilitate change and improve lifestyle behaviors [56]. Further studies are needed that compare a sequential and simultaneous condition for a group that receives general information or no information at all.

Regarding the overall lifestyle behavior changes, no differences were found between the sequential and the simultaneous condition. This is in line with the findings Vandelanotte et al. [26] reported in their study aimed at lowering fat intake and increasing physical activity. We assume that only those respondents with the highest motivational level to change remained in our study at follow-up. Therefore, it might be that no differential effects were found between the sequential and simultaneous condition.

When analyzing the separate behaviors, the largest changes were found for smoking cessation, followed by lower alcohol intake and increased fruit consumption. However, these findings were only partly replicated in the sensitivity analyses. The results of the sensitivity analyses supposed that the effect on the overall risk factor change can mainly be ascribed to changes in fruit consumption, vegetable consumption, and alcohol intake, and probably in tobacco use. Hence, no firm conclusion can be drawn with regard to the differential effects on separate lifestyle behaviors. Further research is needed to investigate whether the optimum tailoring strategy (i.e., a sequential, simultaneous, or even single behavior approach) may depend on the behavior(s) being targeted."

22-ii) Highlight unanswered new questions, suggest future research

- "Future studies should examine the number of behaviors that can be addressed in a multiple behavior change intervention without overloading the respondents [70]."

- "Further studies are needed that compare a sequential and simultaneous condition for a group that receives general information or no information at all."

- "Further research is needed to investigate whether the optimum tailoring strategy (i.e., a sequential, simultaneous, or even single behavior approach) may depend on the behavior(s) being targeted."

Other information

23) CONSORT: Registration number and name of trial registry

Dutch Trial Register NTR2168

24) CONSORT: Where the full trial protocol can be accessed, if available

Schulz DN, Kremers SP, Van Osch LA, Schneider F, Van Adrichem MJ, De Vries H. Testing a Dutch web-based tailored lifestyle program among adults: a study protocol. BMC Public Health 2011;11:108. PMID: 21324181

25) CONSORT: Sources of funding and other support (such as supply of drugs), role of funders
"This study was funded by ZonMw, the Netherlands Organization for Health Research and Development (grant number: 120610012). Intervention development and data analysis took place at Maastricht University. Data collection was done in collaboration with the Regional Health Authorities of the Dutch provinces of North-Brabant (GGD Brabant-Zuidoost; GGD Hart voor Brabant; and GGD West-Brabant) and Zeeland (GGD Zeeland). The authors wish to thank the project partners at the Regional Health Authorities for their assistance in implementing the intervention and recruiting the participants."

X26-i) Comment on ethics committee approval

x26-ii) Outline informed consent procedures
"At the end of this web-based questionnaire, participants were invited to take part in our intervention study. They had to give informed consent and provide their e-mail address for participation and the handling of their data."

X26-iii) Safety and security procedures

X27-i) State the relation of the study team towards the system being evaluated
"Hein de Vries is scientific director of Vision2Health, a company that licenses evidence-based innovative computer-tailored health communication tools. No other authors reported any conflicts of interest."
